# Therapeutic Options for COVID-19: A Review

**DOI:** 10.7759/cureus.10480

**Published:** 2020-09-16

**Authors:** Rishita Pujari, Mary V Thommana, Brisandi Ruiz Mercedes, Ayna Serwat

**Affiliations:** 1 Internal Medicine, Independent Researcher, New York, USA; 2 Ophthalmology, Jawaharlal Nehru Medical College, Belagavi, IND; 3 Medicine, K.J. Somaiya Medical College and Hospital, Mumbai, IND; 4 Internal Medicine, Independent Researcher, Miami, USA; 5 Epidemiology and Public Health, University of Miami Miller School of Medicine, Miami, USA; 6 Infectious Diseases, Independent Researcher, Greenbelt, USA; 7 Infectious Diseases, Hospital Aristides Fiallo Cabral, La Romana, DOM; 8 Internal Medicine, Independent Researcher, Collierville, USA

**Keywords:** covid-19, sars-cov-2, 2019 n-cov, coronavirus, antiviral drug, remdesivir, treatment

## Abstract

An acute respiratory disease caused by a novel coronavirus [severe acute respiratory syndrome coronavirus 2 (SARS-CoV-2), previously known as 2019-nCoV], the coronavirus disease 2019 (COVID-19) was first detected in Wuhan, China. Since then, the virus has spread rapidly worldwide leading to a global public health crisis. Due to its devastating effect on public health, it is crucial to identify a viable therapeutic option to mitigate the damage the disease causes. In spite of various governments implementing aggressive global lock-down and quarantine protocols, the number of cases continues to follow an upward trend. At present, the therapeutic strategies are supportive or preventative, focusing on reducing transmission. Given the gravity of the situation, we aim to explore the drugs that have been tried so far and their efficacy when applied in clinical trials. Since newer interventions would take months to years to develop, by looking at the pool of existing therapeutic options, including remdesivir (RDV), plasma exchange or cytapheresis, hydroxychloroquine, baricitinib, and lopinavir (LPV), we have tried to detail the principles behind their use to treat COVID-19, current application, and adverse effects. Many coronaviruses have a highly mutable single-stranded RNA genome and hence discovering new drugs against the virus is going to be challenging owing to the possible viral genetic recombination. Extensive research is still needed to safely advocate the efficacy of the currently available therapeutic options.

## Introduction and background

Today, the entire world has come to a standstill, grappling with a common enemy. Although we have had numerous outbreaks of diseases in the past, including Ebola, cholera, Middle East respiratory syndrome (MERS), the novel coronavirus pandemic has undoubtedly proven to be a tougher challenge leading to millions of cases and a staggering number of deaths. An outbreak that was presumably first detected in a province in China in late December has found its way into almost every part of the world, leading to a global health crisis. Having a better understanding of the virus through various studies will give us an opportunity to hopefully mitigate the severity of this crisis.

The specific symptomatology of coronavirus disease 2019 (COVID-19) has been challenging to define with the emergence of new symptoms with time. The most common clinical manifestations of COVID-19 include fever, cough, fatigue, dyspnea, sore throat, and headache. Multi-systemic symptoms like anosmia, diarrhea, and vomiting have also been reported [1]. The most common patterns on chest CT imaging in these patients include ground-glass opacity (56.4%) and bilateral patchy shadowing (51.8%). Laboratory findings include lymphocytopenia in 83.2% of patients, thrombocytopenia in 36.2%, and leukopenia in 33.7% people [2]. Severe acute respiratory syndrome coronavirus 2 (SARS-CoV-2) is a betacoronavirus belonging to the *Orthocoronavirinae* subfamily, which also includes severe acute respiratory syndrome coronavirus (SARS-CoV) that caused the severe acute respiratory syndrome (SARS) outbreak in 2002 and the Middle East respiratory syndrome coronavirus (MERS CoV), which caused the MERS outbreak in 2012. The severity of the SARS-CoV-2 infection, when compared to that of SARS and MERS, shows the potential of these viruses to adapt and evolve according to the changing environment [3].

SARS-CoV-2 is a single-stranded, positive-sense, enveloped RNA virus whose genome encodes a number of structural proteins, non-structural proteins, and accessory proteins. The nonstructural proteins [3-chymotrypsin-like protease (3CLpro), papain-like protease (PLpro), helicase, and RNA-dependent RNA polymerase (RdRp)] and the structural protein (spike protein) play an important role in viral pathogenesis and hence they are being recognized as potential targets for drug therapy [4]. The knowledge of the structural components of the virus including its replication and the metabolic pathway has provided an opportunity to identify various drug target areas.

With the rapidly escalating situation worldwide, there are multiple approaches to finding potential therapeutic options as rightly explained by Lu et al. [5]. The first approach would be to look into the pool of existing antiviral drugs by using standard assays. This can help to analyze the effects of these drugs on viral replication. Several drugs have been identified using this method, which includes interferon I (interferon alpha, beta, kappa, lambda, epsilon, etc) and interferon II (interferon-gamma). Since the pharmacokinetic and pharmacodynamic properties and also the side effect profile of these drugs are already known, these drugs have a relative advantage. However, their efficacy against coronaviruses remains unknown as yet [5].

Another approach involves looking through the existing compounds and testing their efficacy for antiviral properties including information about transcription characteristics in different cell lines [5]. These compounds include drugs that alter the neurotransmitter regulation, estrogen receptors, kinase signal transduction, protein processing, and DNA.

A third approach is to redevelop new drugs to act specifically against individual coronaviruses. This would require a thorough understanding of the genomics and structural characteristics of the virus. This includes small interfering RNA (siRNA) molecules or enzymes that will target specific viral enzymes, inhibition of host cell protease, or host viral endocytosis. Although this seems an effective option because of these drugs' specific anti-coronavirus properties, there is limited data regarding the safety profile as well as the pharmacodynamic and pharmacokinetic properties of these drugs. Hence, it would take a long time to prove the efficacy and reliability of these drugs in patients.

A combination of these approaches can help us to evolve treatment options that we can broadly classify into being virus-based or host-based. In this review article, we aim to explore the drugs that have been tried so far and their efficacy when applied in clinical trials. Also, we attempt to explore emerging drug options that look promising.

Genetic characteristics

Coronavirus is classified into four types based on the genomic structure: Alphacoronavirus (AlphaCoV), Betacoronavirus (BetaCoV), Deltacoronavirus (DeltaCoV), and Gammacoronavirus (GammaCoV). As of today, seven human CoVs (HCoV) infecting human species have been identified. BetaCoVs of A lineage (HCoV-OC43 and HCoV-HKU1) and AlphaCoVs (HCoV-229E and HCoV-NL63) can cause the common cold and upper respiratory infections in immunocompetent individuals while lower respiratory infections can occur in immunocompromised people. Other human CoVs, BetaCoVs of the B and C lineage (SARS-CoV, SARS-CoV-2, and MERS-CoV respectively) cause epidemics involving the respiratory and extra-respiratory systems [6].

Genetic characterization has exhibited that bats and rodents are the chief genetic sources of AlphaCoVs and BetaCoVs. On the other hand, avian species appear to be the major genetic sources of DeltaCoVs and GammaCoVs. The viruses of this large family group can cause neurologic, hepatic, respiratory, and intestinal diseases in multiple animal species, which include bats, cats, camels, cattle, and cases of COVID infection have also been detected in feline families in the New York Zoo. SARS-CoV-2 RNA genome contains 29891 nucleotides, which encode for 9860 amino acids, and it has a diameter of 60-140 nm. The genome of SARS-CoV-2 was found to have 89% nucleotide identity with bat SARS-like-CoVZXC21 and 82% with that of human SARS-CoV. Information from these genomic analyses indicates the possibility of SARS-CoV-2 having evolved from a strain found in bats [6]. Although it has been suggested that SARS-CoV2 originated from bats, the role of pangolins acting as intermediate hosts is also being considered. A study has revealed that Pangolin-CoV was found to be 91.02% identical to SARS-CoV-2 and 90.55% identical to BatCoV. Therefore, apart from BatCoV, Pangolin-CoV is most closely related to SARS CoV2 and it could be a natural reservoir of SARS-CoV-2-like CoVs [7]. Thereafter, the virus is presumed to be transmitted to humans, who are the final hosts. However, there is no definitive conclusion about it.

Direct exposure to the Huanan Seafood Wholesale Market of Wuhan was linked to the first few cases of COVID-19, suggesting possible animal to human transmission. But the subsequent cases reported did not follow this mode of transmission. Therefore, the likelihood of human to human transmission was considered. Although symptomatic individuals are the most important source of COVID-19 spread, recent studies also suggest the probability of asymptomatic individuals transmitting the virus [6]. According to multiple studies, the incubation period of the virus ranges from three to seven days, but in some cases, it can take up to two weeks for the symptoms to develop. The main mechanism behind the transmission of this virus is believed to be through respiratory droplets generated during coughing and sneezing, similar to other viral respiratory conditions. Another possible mode of transmission is through exposure to a high concentration of aerosols in closed spaces. Hence, based on multiple studies, close contact between individuals is essential for SARS-CoV-2 to spread [6].

Coronavirus replication and pathogenesis

**Figure 1:**
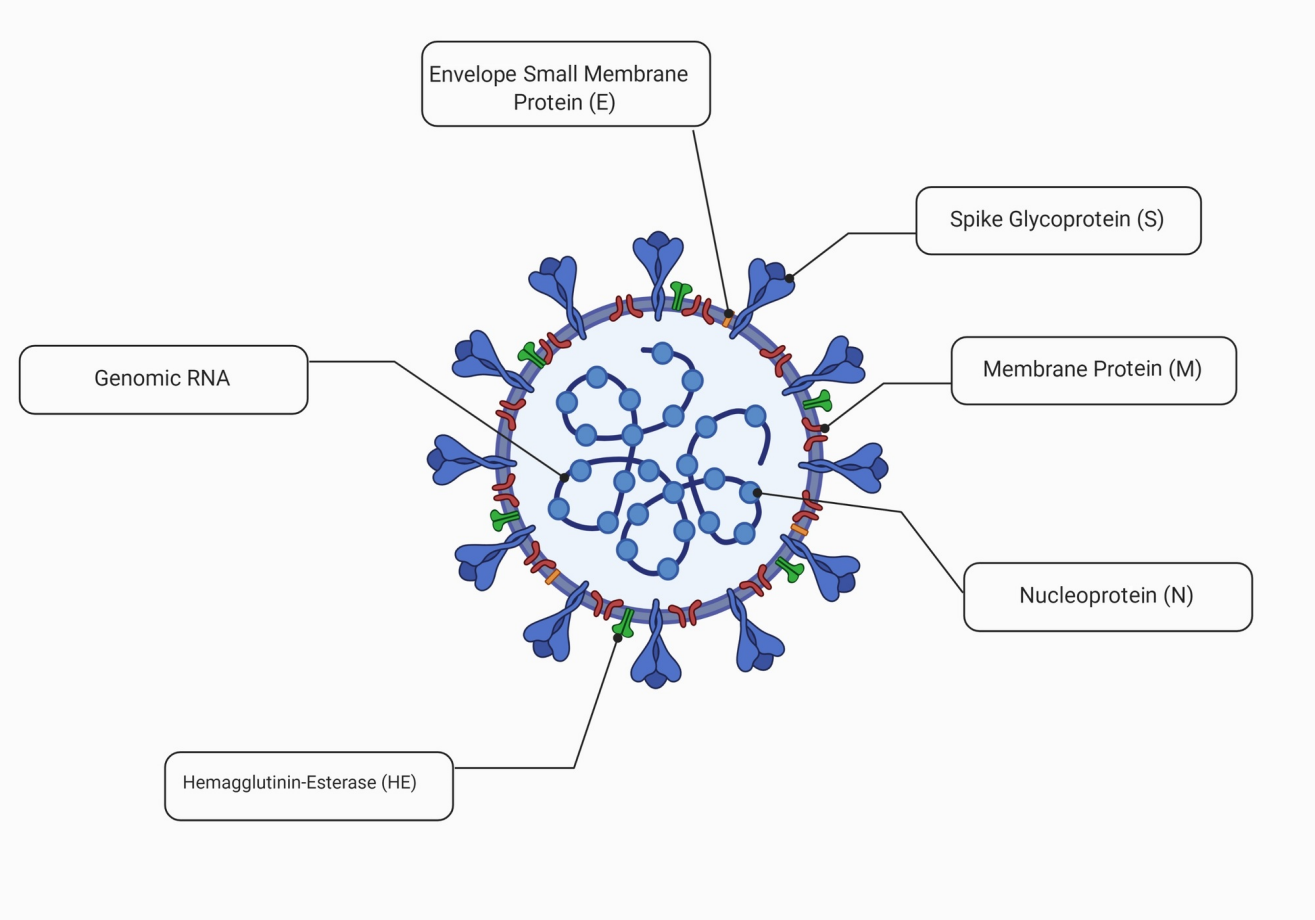
Important structural components of SARS-CoV-2* *[11] RNA: ribonucleic acid; SARS-CoV 2: severe acute respiratory syndrome coronavirus 2

Several studies have reported that CoVs have four structural proteins, which are spike (S), membrane (M), envelope (E), and nucleocapsid (N) [8], as shown in Figure 1. Angiotensin-converting enzyme 2 (ACE2) receptors are located in different organs of the human body, the majority of which are found in the lungs (type 2 pneumocytes, tracheal, and bronchial epithelial cells, macrophages). These ACE2 receptors serve as entry points for SARS-CoV, which is followed by its replication. ACE2 genes are present on the X chromosome and it is found to be expressed in greater proportions in the Asian population than Caucasian and African-American populations [9]. Since the binding of the SARS-CoV-2 spike (S) glycoprotein and ACE2 receptor is a critical step for virus entry, it is under extensive study. Studies have shown that BetaCoV receptors in human cells that express ACE2 have demonstrated increased entry of SARS-CoV-2 [10].

The spike protein is composed of two subunits, S1 and S2, which are cleaved by receptor transmembrane serine protease 2 (TMPRSS2), a member of the hepsin/TMPRSS subfamily [9]. The receptor-binding domain (RBD) in S1 mediates attachment of the virus with the host cell and cellular tropism, while S2, with the help of two tandem domains, heptad repeats 1 (HR1) and heptad repeats 2 (HR2), plays a role in virus-cell membrane fusion. Thereafter, the viral genomic RNA that is released in the cytoplasm translates two different polyproteins, protein phosphatase 1a/protein phosphatase 1b (pp1a/pp1b). These polyproteins encode non-structural proteins forming replication-transcription complex (RTC) in double-membrane vesicles. The replication of RTC proceeds continuously and results in the formation of a subset of genomic RNAs, which is responsible for encoding accessory proteins and structural proteins. The newly formed genomic RNA, along with nucleocapsid proteins, envelopes glycoproteins, endoplasmic reticulum, and Golgi bodies congregate and result in the formation of viral particle buds, which then attach to the plasma membrane resulting in the release of the virus [8].

Pneumonia, which is one of the common complications seen in patients with COVID-19, follows a complex pathogenic mechanism. The virus generates an excessive immune response within the host, also known as a "cytokine storm". Cytokines such as the tumor necrosis factor α (TNF-α), interleukin-1-beta (IL-1β), interleukin-8 (IL-8), interleukin-12 (IL-12), interferon-gamma inducible protein (IP10), macrophage inflammatory protein 1A (MIP-1a), and monocyte chemoattractant protein 1 (MCP1) are released, resulting in the pathogenic cascade of the disease that leads to extensive tissue damage with dysfunctional coagulation. Of these, interleukin-6 (IL-6), which is produced mostly by activated leukocytes, seems to play the primary role in this cascade. IL-6 promotes the differentiation of B lymphocytes and stimulates the production of acute-phase proteins. Cytokine release syndrome (CRS) is an acute systemic inflammatory syndrome that is mediated by IL-6. It is characterized by fever and multiple organ dysfunction. Upon binding of SARS-COV-2 with the toll-like receptor (TLR), there is a release of pro-IL-1β, which is further cleaved into its active mature form. IL-1β plays a major role in lung inflammation and fibrosis seen in patients with COVID-19 [6].

With this knowledge of viral replication and pathogenesis, we can explore various possible therapeutic options, which are further discussed in the article.

Pharmacological targets

**Figure 2:**
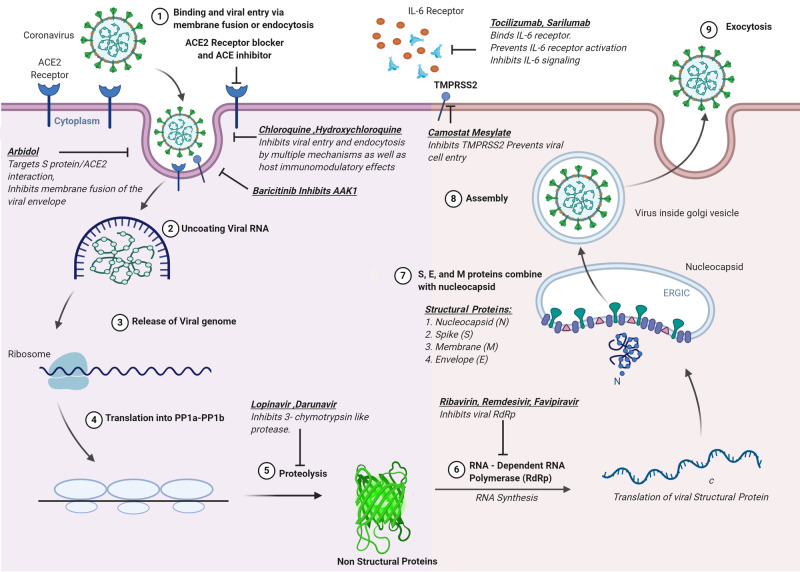
Pharmacological targets for SARS-CoV-2* *[14] SARS-CoV 2: severe acute respiratory syndrome coronavirus 2; ACE2: angiotensin-converting enzyme 2; AAK1: adaptor-associated protein kinase 1; S protein: spike protein; RNA: ribonucleic acid; PP1a: protein phosphatase 1a; PP1b; protein phosphatase 1b; TMPRSS2: transmembrane protease serine 2; IL-6: interleukin-6

Coronavirus replicates by recruiting nonstructural proteins like 3CLpro, PLpro, helicase, and RdRp [12]. These four non-structural proteins are key enzymes within the viral life cycle. In addition, the spike glycoprotein is indispensable for virus-cell receptor interactions during viral entry. These viral proteins were therefore considered as important targets to develop antiviral agents [4]. Therapeutic options in response to the SARS-CoV-2 outbreak are the need of the hour. Here, we discuss the potential for repurposing existing antiviral agents to treat SARS-CoV-2 infection with their site of action as shown in Figure 2, most of which are already moving into clinical trials [4].

*3CLpro and PLpro* 

The polypeptide translation product from the genomic RNA is processed into structural and nonstructural components by the two proteases, namely 3CLpro and PLpro. These proteins are essential for the replication and packaging of the virus. PLpro also acts as a deubiquitinase, which helps to deubiquitylate interferon regulatory factor 3 (IRF3), a host cell protein. It also serves to inactivate the pathway for the nuclear factor κ‐light‐chain‐enhancer of activated B cells (NF‐κB). This ultimately results in immune suppression in the virus-infected host cells. Hence these proteases are potential targets for antiviral agents since they play an important role in viral replication and controlling the host cell. Decoded 3CLpro of SARS-CoV-2 and SARS-CoV exhibit 96% sequence identity, similar to the RdRp protein. Most residues that show variation are found on the protein surface. Hence, in spite of these small variations, small molecule agents that strongly suppress SARS-CoV 3CLpro are anticipated to show the same activity against the SARS-CoV-2 3CLpro. In contrast to 3CLpro, PLpro from the two origins demonstrates only 83% sequence identity. In the case of PLpro, the variations between the two origins occupy the majority of the surface of PLpro. The variations in the amino acid sequence are expected to affect the interaction of PLpro enzymes with their ligands. However, the secondary‐structure components that constitute the active site do not show any variation within the two PLpro proteins. Therefore, an inhibitor developed for the SARS‐CoV PLpro would also possibly work for the SARS-CoV-2 PLpro [13].

Protease Inhibitors (PIs)

PIs like disulfiram, lopinavir (LPV), and ritonavir have shown activity against SARS and MERS. Although disulfiram has been reported to inhibit the PLpro of MERS and SARS in-vitro, it lacks clinical evidence. LPV and ritonavir were considered to suppress the 3CLpro of SARS and MERS and improved clinical outcomes in patients with SARS in a trial. However, its action against the 3CLpro and PLpro of SARS-CoV-2 is still being studied [4]. 

Spike Protein 

Spike protein is a large protein (SARS-CoV-2: 1253 aa; SARS‐CoV: 1273 aa) seen in both SARS-CoV-2 and SARS-CoV. The spike protein from these origins demonstrates a sequence identity of 76%. The majority of the variation has been seen at the N terminus. In the case of SARS-CoV, the S1 site on spike protein contains an RBD, which displays a high interaction with ACE2. It is hypothesized that SARS-CoV-2 also binds to ACE2 to enter the human host cell by engaging this RBD. The RBD from the two origins exhibits a 73.5% sequence identity. It has also been speculated that the interaction between ACE2 and SARS-CoV-2 RBD is weaker than that of SARS-CoV RBD. However, since both domains are highly looped structures, large variations in these two sites will lead to structural rearrangements that will result in even stronger interactions with ACE2. Unpredictability about these interactions will continue to persist until the structure of the SARS-CoV-2-RBD-ACE2 complex is extensively studied. Before any experimental results are available, any claims about the weaker binding of the SARS-CoV-2 RBD toward ACE2 compared to that of the SARS‐CoV RBD is premature [13]. A promising drug targeting the spike glycoprotein is griffithsin, which is a red-algae-derived protein. It acts by binding to oligosaccharides found on the surface of numerous viral glycoproteins including SARS-CoV spike glycoprotein and HIV glycoprotein 120. Although griffithsin is currently under study for HIV prevention, its potency and efficacy for the treatment or prevention of SARS-CoV-2 should be researched further [4].

## Review

Therapeutic options

Remdesivir

Current applications: remdesivir (RDV) is one of the most important drugs named in the treatment of COVID-19 and is being extensively researched. In 2017, Gilead Sciences developed RDV for the treatment of Ebola virus infection. It is a broad-spectrum drug having antiviral activities against RNA viruses such as SARS-CoV-2, SARS-CoV, and MERS-CoV [12].

Mechanism of action (MOA)*: *RDV (GS-5734), a nucleoside analog, is an RdRp inhibitor. In particular, the triphosphate form of RDV (RDV-TP) has shown to inhibit the RdRp of the virus. The host metabolizes RDV into the active triphosphate form. The primary mechanism of inhibition is the incorporation of RDV-TP into nascent RNA chains by RdRp. This causes delayed RNA chain termination during the process of viral replication [15]. 

Principle behind use: RDV has been shown to have activity against varying coronaviruses in the airway epithelial cells in humans [12]. Preliminary results of the Adaptive COVID-19 Treatment Trial (ACTT-1) suggest that a 10-day course of RDV was more effective than a placebo in the treatment of hospitalized patients with COVID-19. The benefit was seen in terms of shortened recovery time (11 days versus 15 days). On May 1, 2020, the US Food and Drug Administration (FDA) issued an Emergency Use Authorization (EUA) for RDV for the treatment of adults and children hospitalized with severe suspected or laboratory-confirmed COVID-19, which was later expanded to include all adult and pediatric patients hospitalized with suspected or laboratory-confirmed COVID-19, and for whom the use of an intravenous (IV) agent is clinically appropriate [16]. 

Patient selection and treatment initiation: the selection of patients and treatment initiation are as follows:

- Empiric treatment can be initiated in suspected COVID-19 patients whose laboratory results are pending. 

- Adult and pediatric patients (older than 28 days of age) must have an estimated glomerular filtration rate (eGFR) determined, and full-term neonates (at least seven days to less than or equal to 28 days of age) must have serum creatinine determined before dosing and daily while receiving RDV.

- Hepatic laboratory testing should be performed in all patients before starting RDV.

- RDV can be used at any time after the onset of symptoms in hospitalized patients [16].

Dosage: at present, for adults, the recommended dosage is a single loading dose of 200 mg on day one followed by a once-daily maintenance dose of 100 mg from day two. For pediatric patients weighing 3.5 kg to less than 40 kg, RDV should be injected as 100 mg, lyophilized powder only. For pediatric patients weighing 40 kg and higher, the adult dosage regimen of a single loading dose of 200 mg on day one followed by once-daily maintenance doses of 100 mg from day two is recommended [16]. On June 22, 2020, Gilead announced that the company is about to start human trials on an inhaled version of the drug for easier administration outside the hospital in the early stages of the disease [17].

Adverse effects and challenges: as for safety, according to Gilead Sciences, infusion-related and anaphylactic hypersensitivity reactions were seen following RDV administration, which could be avoided with slowing down the infusion rates. RDV is contraindicated in patients with known hypersensitivity reactions. Elevated transaminases were observed in patients with COVID-19 and, therefore, RDV should not be used in patients with alanine transaminase (ALT) levels of ≥5x ULN (upper limit of normal). The drug should be discontinued if patients develop ALT of ≥5x ULN or ALT elevation accompanied by signs or symptoms of liver inflammation or increasing conjugated bilirubin, alkaline phosphatase, or INR (international normalized ratio). According to reports, the antiviral effects of RDV is reduced if co-administered with chloroquine phosphate or hydroxychloroquine sulfate [16]. Multiple clinical trials are ongoing on the use of RDV for the treatment of COVID-19.

Therapeutic Apheresis (Plasma Exchange or Cytapheresis)

Current applications: at present, plasma exchange is being used as a therapeutic option in various conditions like myasthenia gravis, Guillain-Barré syndrome, chronic inflammatory demyelinating polyneuropathy, fulminant Wilson's disease, hypergammaglobulinemia manifested with hyperviscosity, thrombotic thrombocytopenic purpura, complement-mediated thrombotic microangiopathy, and Goodpasture syndrome [18].

MOA: therapeutic plasma exchange is “a therapeutic procedure in which blood of the patient is passed through a medical device which separates out plasma from other components of blood. The plasma is removed and replaced with a replacement solution such as a colloid solution (eg, albumin and/or plasma) or a combination of crystalloid/colloid solution” [18]. Immune (‘convalescent’) plasma contains antibodies that exert their therapeutic effects through multiple mechanisms. These include binding of the antibody to the pathogen and thereby negating its infectivity directly or through antibody-mediated pathways like complement activation and antibody-dependent cellular cytotoxicity [19]. The efficacy of convalescent blood products in cases of viral infections was first noted during Spanish influenza. Thereafter, the possibility of the usage of convalescent plasma for prevention and/or treatment gathered interest during the West African Ebola outbreak. Various other infections caused by West Nile Virus, MERS-CoV, and SARS-CoV-1 have also been considered as potential targets for plasma exchange [20].

Principle behind use: severe cases of COVID-19 are characterized by a cytokine storm defined as a sudden production of cytokines, such as IL-2, IL-7, IL-10, granulocyte colony-stimulating factor (G-CSF), MCP1, MIP-1a, and TNF-α, secondary to the upregulation of the inflammatory process in COVID-19 [21]. As suggested by Lin et al. [22], the viral attack occurs in two stages. In the first stage, the virus attacks the organs that express ACE2 receptors while the second attack occurs 7-14 days after symptom onset. In the early phases, the virus causes a reduction in B lymphocyte and IL-6 counts, which can have an impact on antibody production. The lymphocyte count continues to drop over the course of the disease along with a rise in inflammatory cytokines. Therefore, an ideal therapeutic agent should enhance the immune function of the patient along with slowing down the production of inflammatory cytokines [20]. The use of plasma exchange provides a potentially promising option until other treatment options and vaccines are being explored [20]. Plasma exchange thus appears to be a promising option for effective management of COVID-19 patients.

Dosage: the average dosage used in multiple studies has been around 200 mL with a few patients requiring multiple infusions [19]. FDA has laid down certain guidelines regarding patient eligibility for treatment with plasma exchange: 

1. Laboratory-confirmed COVID-19

2. Severe or immediately life-threatening COVID-19; for example, severe disease is defined as the presence of one or more of the following:

 - Shortness of breath (dyspnea),

 - Respiratory frequency of ≥30/min,

 - Blood oxygen saturation of ≤93%,

 - Partial pressure of arterial oxygen to fraction of inspired oxygen ratio of <300,

 - Lung infiltrates of >50% within 24 to 48 hours.

Life-threatening disease is defined as the presence of one or more of the following:

 - Respiratory failure,

 - Septic shock,

 - Multiple organ dysfunction or failure.

3. Informed consent provided by the patient or healthcare proxy [23].

Adverse effects and challenges: as with any transfusion, plasma exchange carries a risk of transfusion-transmissible infections, the incidence of which is very low in the US. Other noninfectious risks include allergic transfusion reactions, transfusion-associated circulatory overload (TACO), and transfusion-related acute lung injury (TRALI). TRALI is especially important in critically ill patients due to COVID-19. These patients may have underlying acute respiratory distress syndrome (ARDS), which could be worsened by TRALI. Volume overload (TACO) has the potential to precipitate pulmonary edema, especially in older adults, small children, and those with preexisting cardiac disease. Anaphylactic reactions may occur in patients with Immunoglobulin A (IgA) deficiency and antibodies to IgA [19]. A potential challenge that can affect the effectiveness of plasma exchange as a therapeutic option is the plasma antibody titer, which can vary among donors. Hence ensuring a high antibody titer specific for SARS-CoV-2 is crucial for optimum results. Also, the use of convalescent plasma has to be researched further due to the multidisciplinary approach in critically ill patients who are frequent recipients of plasma exchange [20].

Chloroquine

Current applications: the primary indication for chloroquine and hydroxychloroquine is to prevent or treat malaria and also in the treatment of several infections like HIV, Q fever, Whipple disease, and fungal infections. Other indications of these drugs include immunological and rheumatologic disorders like systemic lupus erythematosus (SLE), antiphospholipid antibody syndrome, rheumatoid arthritis, and Sjögren's syndrome. Chloroquine and hydroxychloroquine have been found to have anti-inflammatory, immunomodulating, anti-infective, antithrombotic, and metabolic effects. They also have an anti-tumoral effect because of their strong antiproliferative, antimutagenic, and inhibiting autophagy capacities [24].

MOA*:* chloroquine and hydroxychloroquine have several mechanisms of action. Since these drugs are weak bases, they increase endosomal pH in host intracellular organelles resulting in inhibition of autophagosome-lysosome fusion and inactivation of enzymes required for viral replication [25]. Chloroquine demonstrates broad-spectrum antiviral activity against human coronavirus HCoV-O43 and orthomyxoviruses by interfering with sialic acid biosynthesis since these viruses use sialic acid moieties as receptors. This is due to inhibition of quinone reductase, structurally similar to UDP-N-acetylglucosamine 2-epimerases, which is a key enzyme in the synthesis of sialic acids. Chloroquine exerts a potent anti-SARS-CoV-1 effect in vitro, which is due to a deficit in the glycosylation of a cell surface receptor, the ACE2 on Vero cells. Chloroquine has an effect on the immune system by its action on cell signaling and the regulation of pro-inflammatory cytokines. Cell activation by mitogen-activated protein kinase (MAPK) signaling is an important step in the viral replication cycle. Chloroquine prevents phosphorylation of p38 MAPK in Tamm-Horsfall protein 1 (THP-1) cells as well as caspase-1, which was demonstrated in a model of HCoV-229 coronavirus. Chloroquine, being an immunomodulatory agent, induces inhibition of IL-1, IL-6, TNF-α in immune cells [26].

Principle behind use: chloroquine and hydroxychloroquine are 4-aminoquinolines that are under trial for the treatment of COVID-19. Currently, many clinical trials are ongoing worldwide for chloroquine, hydroxychloroquine, or these two in combination with other drugs. Chloroquine has been found to affect glycosylation of ACE2, the receptor that SARS-CoV-2 uses to enter cells [25]. Several in-vitro studies have been done in which chloroquine has acted against both RNA and DNA viruses. Some of those RNA viruses include HIV, influenza A and B virus, Zika virus, and DNA viruses include the hepatitis B virus and herpes simplex virus. Coronavirus being an RNA virus, chloroquine has demonstrated in vitro inhibition of HCoV-229E replication in epithelial lung cultures. In another study, it was concluded that infections in recently born mice with the HCoV-O43 coronavirus could have been prevented by administering chloroquine through the mother's milk. These studies also exhibited an antiviral effect of chloroquine on a recombinant HCoV-O43 coronavirus [26]. Currently, there are several ongoing clinical trials to test the efficacy of chloroquine in combination with multiple drugs. On April 27, 2020, the FDA issued a EUA for the emergency use of chloroquine to treat adults and adolescents who weigh 50 kg or more and are hospitalized for COVID-19 when a clinical trial is unavailable or participation is not feasible. The WHO had also added the drug to its Solidarity trial. On June 15, 2020, this was revoked since the drug did not decrease the probability of death or expedite recovery [27].

Dosage: in accordance with various guidelines, the effective dose of chloroquine for the treatment of COVID-19 infection has been found to be 500 mg twice daily for 10 days [28]. According to a study, for pre-exposure prophylaxis, an 800 mg loading dose followed by 400 mg twice or three times weekly is required. In a post-exposure prophylaxis setting, 800 mg loading dose followed in six hours by 600 mg, then 600 mg daily for four more days is required. These doses are higher than recommended in cases of malaria prophylaxis and clinical trials are needed to establish safety and efficacy [29].

Adverse effects and challenges: there have been some concerns regarding the safety of hydroxychloroquine in high doses for treatment in critically ill patients with COVID-19 [30]. Hydroxychloroquine can cause serious cutaneous adverse reactions, fulminant hepatic failure, and ventricular arrhythmias like QTc (corrected Q-T interval) prolongation especially when prescribed with azithromycin [25]. Routine electrocardiogram (ECG) is therefore recommended before starting these drugs. Drug interactions should be taken into consideration, especially the administration of drugs that cause QTc prolongation such as antiarrhythmic, antidepressants, anti-psychotics, antihistamine, ondansetron, and moxifloxacin; LPV/ritonavir and RDV should be avoided. Administration of hydroxychloroquine and chloroquine requires frequent monitoring of complete blood count, which includes red blood cells (RBC), white blood cells (WBC) and platelet counts, measurement of serum electrolytes, serum blood glucose level (since hydroxychloroquine can cause hypoglycemia), hepatic [liver function tests (LFTs)] and renal functions. Other contraindications include hypersensitivity reactions, retinopathy, porphyria, epilepsy, pre-existing maculopathy, glucose-6-phosphate dehydrogenase (G6PD) deficiency, recent myocardial infarction (MI), and QTc of >500 msec [28].

Lopinavir

Current application: lopinavir (LPV) is an HIV-1 protease inhibitor administered in fixed-dose combination with ritonavir (LPV/r). Ritonavir is a potent cytochrome P450 3A4 (CYP3A4) inhibitor, which further potentiates the pharmacokinetic and pharmacodynamic properties of LPV [12]. This combination has been found to effectively reduce viral load in HIV patients leading to an interest in its possible role in treating viruses with similar machinery [12].

MOA: as mentioned above, proteases are vital enzymes that play an important role in viral replication, maturation, and assembly process. Inhibition of these protease enzymes can help to effectively limit the spread of the virus in the host cells. LPV/r interferes with HIV protease, resulting in inhibition of HIV replication by causing dysregulation of structural and functional proteins in the virus core, contributing to the generation of immature and noninfectious virus particles [31].

Principle behind use: all coronaviruses like SARS-CoV, MERS CoV, and SARS-CoV-2 are RNA viruses like HIV. According to various studies, the nonstructural protein of coronavirus, especially the main proteases or 3CLpro, is involved in the proteolytic processing of the replicase polyprotein, which in turn is vital for viral replication and maturation [32]. LPV/r has shown promising results in vitro studies in the inhibition of 3CLpro found in nCoV, thus possibly limiting spread in host cells. LPV showed an antiviral effect against the SARS-CoV-2 virus in Vero E6 cells with the estimated EC50 (half-maximal effective concentration) at 26.63 μM [33].

Dosage: based on numerous clinical studies, an LPV/r dosage of 400 mg/100 mg twice daily has been found to be effective as an adjunctive therapy [34].

Adverse effects and challenges: adverse side effects include intolerable gastrointestinal toxicity and diarrhea. There may also be an increased risk of hepatotoxicity, hence close laboratory monitoring may be needed. With ritonavir being a CYP3A4 inhibitor, it is also a moderate inhibitor of p-glycoprotein (P-gp), organic anion transporter (OATP1B1), OATP1B3, and is an inducer of uridine diphosphate-glucuronosyltransferases (UGT). A sizable majority of patients with COVID- 19 requiring ICU admission are elderly with various comorbidities and polypharmacy. Therefore, considerable attention should be paid to any possible drug interactions in these patients [12]. Another area of concern, as aptly described by Li et al., is that while HIV protease belongs to the aspartic protease family, the two coronavirus proteases are from the cysteine protease family. In addition to this, HIV protease inhibitors were specifically designed to fit the C2 symmetry in the catalytic site of the HIV protease dimer, but the coronavirus proteases lack this C2 symmetric site. So the potency and efficacy of LPV/r remain debatable [4]. Despite data showing in vitro effectivity of LPV/r, some studies have revealed no benefit in patients treated with LPV/r when compared with standard care [34].

Monoclonal Antibodies

Current applications: tocilizumab is a recombinant humanized monoclonal antibody directed against the IL-6 receptor (IL-6R). It has been approved by the FDA for the treatment of severe chimeric antigen receptor (CAR) T cell-induced CRS, giant cell arteritis, rheumatoid arthritis (RA), and polyarticular or systemic juvenile arthritis [35,36]. 

MOA: as mentioned above, excess stimulation of the immune response leads to an exaggerated release of IL-6, which is responsible for a series of events resulting in organ damage and worsening of the overall clinical condition of the patient. Tocilizumab inhibits IL-6-mediated signaling by binding to both soluble and membrane-bound IL-6R, ultimately causing effective downregulation of the immune system [36].

Principle behind use: in a majority of seriously ill patients of COVID 19, the leading cause of death has been found to be ARDS and multi-organ system failure. This has been linked to an exaggerated immune response leading to the heightened synthesis of inflammatory cytokines and chemokines like IL-6, IL-1β, IP10, and MCP1. This leads to a condition of severe systemic inflammatory response also known as CRS [37]. In these patients, in spite of having diminishing viral loads, the reason for their deteriorating health is the amplified immune response. Hence the answer to a possible therapeutic intervention cannot rely on antiviral alone. IL-6 is a key inflammatory driver in these reactions. Recent guidelines have indicated that IL-6 can be a biomarker indicating worsening of COVID-19 [38]. Hence, anti-IL-6 drugs like tocilizumab can be a promising therapeutic option by downregulating the exaggerated immune response. 

Dosage: the optimal dose and schedule of tocilizumab is not clearly defined [36]. In a few studies, a one-time dose of IV tocilizumab 400 mg showed promising results [39]. However, one of the studies mentioned that a few patients required another dose within 12 hours [39]. This was in addition to other standard therapies including LPV, methylprednisolone, and oxygen therapy.

Adverse effects and challenges: a cautious approach towards the use of tocilizumab is advised since there is limited data on its efficacy and potential toxicity. Its use in patients with tuberculosis (TB) or any active infection is contraindicated. Elevated IL-6 levels have been seen as an important inclusion criterion in these studies [39]. Although no adverse effects were reported in patients in the above-mentioned studies, patients with RA who are on chronic tocilizumab therapy were found to be at an increased risk of infections. Careful laboratory monitoring has been recommended due to treatment-related changes in platelets, neutrophils, and liver enzymes. Other common side effects are upper respiratory tract infections, nasopharyngitis, headache, hypertension, increased ALT, and injection site reactions [35].

Baricitinib

Current use: the Janus kinase-signal transducer and activator of transcription (JAK-STAT) pathway is essential in the immune response to exogenous signals and substances. The JAK family of enzymes is responsible for signal transduction, and JAK inhibitors play a role in inhibiting cytokine release that can lead to cancer cell growth. Baricitinib, a JAK inhibitor, is used in the treatment of some types of malignancies and autoimmune diseases such as RA due to its immunomodulatory effect [40].

MOA: baricitinib is a reversible inhibitor of JAK1 and JAK2, which results in blockage of signal transmission from cytokines or growth receptors. This leads to reduced hematopoiesis and immune cell function. This blockade of signal transmission impedes phosphorylation, which further prevents the activation of signal transducers and activators of transcription. It is postulated that baricitinib could prevent the intracellular movement of viral cells and the development of viral particles due to its affinity for binding to associated protein kinase 1 (AAK1) [12].

Principle behind use: due to the anti-inflammatory action of baricitinib, it was independently hypothesized using artificial intelligence (AI)-algorithms to be useful for the treatment of COVID-19 infection via proposed anti-cytokine effects and as an inhibitor of host cell viral propagation. As mentioned previously, CRS has been linked to increased morbidity and mortality in COVID-19 patients. Baricitinib has been found to inhibit IL-6-induced MCP1 production from human peripheral blood mononuclear cells [41].

Adverse effects and challenges: lymphopenia and anemia have been identified as potential adverse effects in multiple clinical trials, which can worsen the outcome in severe cases of COVID-19. Baricitinib therapy also increases the levels of creatine kinase, and this elevation is more common in patients in ICU and critically ill. Some studies have reported an increased incidence of respiratory tract infections as high as 16.3% and an incidence of other infectious diseases as high as 29-42%. Co-infection is a significant threat during treatment with baricitinib. There is an increased risk of reactivation of latent infections like TB, hepatitis B, Varicella-zoster, herpes simplex, and Epstein Barr virus strains [42]. As observed in a study, a major concern with the use of anti-inflammatory medications is that they can impede the clearance of viruses, thereby resulting in an increased risk for secondary infections [43]. This risk is further increased in elderly patients or those with an impaired immune system. Also, the biological agents targeting pro-inflammatory cytokines can only inhibit specific inflammatory factors, but the whole cascade is still active and affecting the system. Another concern is that some anti-inflammatory medications such as JAK inhibitors target and hamper interferon-alpha production, which plays a key role in the antiviral endogenous response, and theoretically may not be apt for the inflammatory cytokine storm caused by viruses such as COVID-19. In addition, the efficacy of baricitinib against COVID-19 is still under study [43].

Ribavirin

Ribavirin is a guanosine analog that interferes with viral RNA synthesis by inhibiting viral RdRp as well as messenger RNA (mRNA) capping [44]. Studies have shown that while SARS-CoV, MERS-CoV, and HCoV-OC43 were sensitive to ribavirin in vitro, drug concentrations that significantly impede CoV replication is higher than what can be safely achieved in humans. A reason for the decreased in vitro efficacy of ribavirin was found to be the excision of ribavirin nucleoside analogs by coronavirus proofreading mechanism. Subsequent in vivo testing showed that monotherapy with ribavirin showed restricted activity against SARS-CoV in mouse models. However, in primate models, improvement of MERS symptoms was seen when ribavirin was combined with type I Interferons [44]. In a recent study, it was also demonstrated that the SARS-CoV-2 RdRp model is targeted by ribavirin [45].

Angiotensin-Converting Enzyme Inhibitor (ACEI) [Renin-Angiotensin System (RAS) Drugs]

As mentioned under pathogenesis, SARS-CoV-2 enters the cells of our body through ACE2. Some have suggested that the use of ACE inhibitor impairs (ACEI) the ACE/angiotensin-II/angiotensin-1 receptor pathway. which further impedes the integrity of the ACE2/angiotensin 1-7/MAS (MAS-related G protein-coupled receptor). This ultimately could result in decreased production of ACE2, thereby reducing the chances of SARS-CoV-2 entering the cell. However, it has also been argued that ACEI inhibits ACE and decreases the level of angiotensin-1. This in turn through possible negative feedback, upregulates more ACE2 receptors, leading to increased binding sites for SARS-CoV-2. A recent article published in The Lancet mentions that drugs causing an increase in ACE2 receptors are at a higher risk for severe COVID-19 infection [46]. Therefore, the balance of potential benefits and harms from continuing ACEI or angiotensin receptor blockers (ARB) therapy during an acute infection depends on the reason for prescribing. Although these drugs initially sparked interest due to their interaction with ACE receptors, which in turn play a major role in the pathogenesis of COVID infection, they are not being viewed as a potential COVID-19 treatment option. Currently, there are some clinical trials focussing on stopping ACE inhibitors and ARBs for patients with COVID 19. Data on the renin-angiotensin system (RAS) drugs acting on lung-specific expression of ACE2 in human and animal experimental models are lacking. Further studies in humans need to be performed to better understand the relation between SARS-CoV-2 and the renin-angiotensin-aldosterone system (RAAS) pathways.

In Table 1 and Table 2, we have laid out a summary of ongoing randomized control trials for individual and combination drugs. All information regarding the trials has been accessed from the ClinicalTrials.gov database [47].

**Table 1:** Randomized control trials for individual interventions *Due to the vast number of ongoing trials, trials in this chart are limited to those with >500 participants NCT ID: National Clinical Trial Identifier; ACE: angiotensin-converting enzyme; SARS-CoV-2: severe acute respiratory syndrome coronavirus 2; COVID-19: coronavirus disease 2019

Intervention	NCT ID	No. of participants	Duration	Phase	Sponsor
Remdesivir					
Drug: remdesivir; drug: standard of care	NCT04292899	6000	March 2020–May 2020	Phase 3	Gilead Sciences
Drug: remdesivir; drug: standard of care	NCT04292730	1600	March 2020–May 2020	Phase 3	Gilead Sciences
Lopinavir					
Drug: lopinavir/ritonavir	NCT04321174	1220	April 2020–March 2022	Phase 3	Darrell Tan
Tocilizumab					
Drug: tocilizumab	NCT04377750	500	April 2020–May 2021	Phase 4	Hadassah Medical Organization
Drug: tocilizumab; drug: placebo	NCT04335071	100	April 2020–October 2020	Phase 2	University Hospital Inselspital, Berne
Drug: tocilizumab	NCT04403685	150	May 2020–August 2020	Phase 3	Beneficência Portuguesa de São Paulo
Drug: tocilizumab; drug: placebo	NCT04320615	330	April 2020–September 2020	Phase 3	Hoffmann-La Roche
Drug: tocilizumab	NCT04331808	228	March 2020–December 2021	Phase 2	Assistance Publique - Hôpitaux de Paris
Baricitinib					
Drug: baricitinib; drug: placebo	NCT04421027	400	June 2020–September 2020	Phase 3	Eli Lilly and Company
ACEI					
Drug: ACE inhibitor, angiotensin receptor blocker	NCT04353596	208	April 2020–May 2021	Phase 4	Medical University Innsbruck
Plasma apheresis*					
Biological: convalescent plasma	NCT04348656	1200	May 2020–December 2020	Phase 3	Hamilton Health Sciences Corporation
Biological: convalescent plasma; biological: standard donor plasma	NCT04344535	500	April 2020–August 2021	Phase 1, phase 2	Stony Brook University
Biological: SARS-CoV-2 convalescent plasma; biological: plasma from a volunteer donor	NCT04373460	1344	June 2020–January 2023	Phase 2	Johns Hopkins University
Biological: pathogen reduced SARS-CoV-2 convalescent plasma; biological: placebo	NCT04362176	500	April 2020–April 2021	Phase 3	Vanderbilt University Medical Center
Drug: plasma from COVID-19 convalescent patient; drug: human immunoglobulin	NCT04381858	500	May 2020–September 2020	Phase 3	Centenario Hospital Miguel Hidalgo

**Table 2:** Randomized control trials of combination drugs *Due to the vast number of ongoing trials, trials in this chart are limited to those with >1000 participants NCT ID: National Clinical Trial Identifier; SARS-CoV-2: severe acute respiratory syndrome coronavirus 2

Intervention	NCT ID	No. of participants*	Duration	Phase	Sponsor
Combination drugs					
Drug: hydroxychloroquine + intravenous famotidine	NCT04370262	1170	April 2020– April 2021	Phase 3	Northwell Health
Drug: thiazide or thiazide-like diuretics; drug: calcium channel blockers; drug: ACE inhibitor; drug: angiotensin receptor blocker	NCT04330300	2414	March 2020– March 2021	Phase 4	National University of Ireland, Galway, Ireland
Drug: lopinavir/ritonavir; drug: hydroxychloroquine sulfate; drug: losartan; drug: placebos	NCT04328012	4000	April 2020–April 2021	Phase 2, phase 3	Bassett Healthcare
Drug: remdesivir; drug: lopinavir/ritonavir; drug: interferon beta-1A; drug: hydroxychloroquine; other: standard of care	NCT04315948	3100	March 2020–March 2023	Phase 3	Institut National de la Santé Et de la Recherche Médicale, France
Drug: lopinavir/ritonavir; drug: hydroxychloroquine; drug: remdesivir	NCT04330690	2900	March 2020–May 2022	Phase 2	Sunnybrook Health Sciences Centre
Biological: convalescent anti-SARS-CoV-2 plasma; drug: sarilumab; drug: baricitinib; drug: hydroxychloroquine; other: injective placebo; other: oral placebo	NCT04345289	1500	May 2020–June 2021	Phase 3	Thomas Benfield
Drug: lopinavir-ritonavir; drug: corticosteroid; drug: hydroxychloroquine; drug: azithromycin; biological: convalescent plasma; drug: tocilizumab	NCT04381936	12000	March 2020–June 2021	Phase 2, phase 3	University of Oxford
Drug: hydroxychloroquine sulfate tablets; drug: lopinavir/ritonavir oral tablet; drug: hydroxychloroquine sulfate tablets plus lopinavir/ritonavir oral tablets; drug: placebo	NCT04403100	1968	June 2020–November 2020	Phase 3	Cardresearch
Drug: hydroxychloroquine; drug: azithromycin	NCT04334382	1550	April 2020–December 2021	Phase 3	Intermountain Health Care, Inc.
Drug: hydroxychloroquine; drug: azithromycin (Azithro); drug: placebo for hydroxychloroquine; drug: placebo for azithromycin	NCT04358068	2000	May 2020–March 2021	Phase 2	National Institute of Allergy and Infectious Diseases (NIAID)
Drug: hydroxychloroquine; drug: placebo oral tablet	NCT04318444	1600	March 2020–March 2022	Phase 2, phase 3	Columbia University
Drug: favipiravir (3200 mg + 1200 mg); drug: favipiravir (3600 mg + 1600 mg); drug: favipiravir (3200 mg + 1200 mg) combined with hydroxychloroquine; drug: favipiravir (3200 mg + 1200 mg) combined with azithromycin; drug: hydroxychloroquine; drug: hydroxychloroquine combined with azithromycin	NCT04411433	1000	May 2020–July 2020	Phase 3	Ministry of Health, Turkey
Drug: hydroxychloroquine - daily dosing; drug: hydroxychloroquine - weekly dosing	NCT04341441	3000	April 2020–April 2021	Phase 3	Henry Ford Health System
Drug: hydroxychloroquine	NCT04363827	2300	May 2020–March 2021	Phase 2	Istituto Scientifico Romagnolo per lo Studio e la Cura dei Tumori
Drug: hydroxychloroquine sulfate; drug: ascorbic acid	NCT04328961	2000	March 2020–October 2020	Phase 2, phase 3	University of Washington
Drug: hydroxychloroquine; drug: placebo oral tablet	NCT04334148	15000	April 2020–July 2020	Phase 3	Adrian Hernandez
Drug: emtricitabine/tenofovir disoproxil; drug: hydroxychloroquine; drug: placebo: emtricitabine/tenofovir disoproxil placebo; drug: placebo: hydroxychloroquine	NCT04334928	4000	April 2020–July 2020	Phase 3	Plan Nacional sobre el Sida (PNS)
Drug: hydroxychloroquine; drug: placebo	NCT04325893	1300	April 2020–September 2020	Phase 3	University Hospital, Angers
Drug: hydroxychloroquine; drug: placebo oral tablet	NCT04421664	1500	March 2020–August 2020	Phase 3	McGill University Health Centre/Research Institute of the McGill University Health Centre
Dietary supplement: vitamins; drug: hydroxychloroquine; drug: imatinib; drug: favipiravir; drug: telmisartan	NCT04356495	1057	July 2020–January 2021	Phase 3	University Hospital, Bordeaux
Drug: hydroxychloroquine; other: placebo	NCT04308668	3000	March 2020–May 2020	Phase 3	University of Minnesota
Other: placebo; drug: remdesivir; drug: baricitinib	NCT04401579	1032	May 2020–August 2023	Phase 3	National Institute of Allergy and Infectious Diseases (NIAID)

Emerging options

There are many emerging options being tried for the treatment of COVID-19. We have listed a few important ones. 

Stem Cell Therapy 

Stem cell therapy has sparked interest in many researchers as they are resistant to tissue damage, promote tissue repair, and have immunomodulatory effects. Treatment with mesenchymal stem cells (MSCs) has been found to inhibit the overactivation of the immune system and, by improving the lung microenvironment, they promote endogenous repair [48]. More than 70 clinical trials are registered to assess the properties of MSCs, whereas 17 completed trials have confirmed their immunomodulatory and anti-inflammatory properties [47]. (NCT04313322, NCT04315987, NCT04302519, NCT04288102, NCT04273646, NCT04252118, NCT04299152, NCT04269525, NCT04276987,NCT04389450, NCT04367077).

Neutrophil Extracellular Trap (NET) Therapeutics

NETs are web-like DNA proteins expelled from the neutrophils that trap pathogens. Excessive NET formations can trigger a cascade of inflammatory reactions that destroy surrounding tissues, facilitating microthrombosis leading to permanent damage to pulmonary and other systems. The coagulation abnormality seen in infected patients is most likely due to the widespread inflammatory response as the virus does not appear to have intrinsic procoagulant effects. Early reports from the first 99 patients hospitalized in China showed that 5% had elevated prothrombin time (PT), 6% had elevated activated partial thromboplastin time (aPTT), 36% had elevated D-dimer, with increased biomarkers of inflammation such as IL-6, Erythrocyte sedimentation rate (ESR) and c-reactive protein (CRP). Drugs like neutrophil elastase inhibitors, protein arginine deiminase 4 (PAD4) inhibitors, gasdermin D inhibitors, deoxyribonuclease (DNase), colchicine, and IL-1β are some drugs targeting NETs while some are still under development. Of these, colchicine and anakinra are under study [49]. (ClinicalTrials.gov identifiers: NCT04324021, NCT04330638, NCT02735707, NCT04326790, NCT04328480, NCT04322565, NCT04322682, NCT04366232, NCT04424056, NCT04412291, NCT04362943, NCT04362111, NCT04360980, NCT04363437, NCT04350320, NCT04355143, NCT04367168, NCT04326790,NCT04359654) [47].

Arbidol

Arbidol is an antiviral that has been approved in China and Russia for treating influenza, SARS, and Lassa viruses. It blocks virus replication by inhibiting the fusion of the lipid membrane of the virus with the host cells. A study with Arbidol treatment showed it to be possibly superior to LPV/ritonavir in treating COVID-19 because the viral load was undetectable after administration of Arbidol in patients [50]. (ClinicalTrials.gov identifiers: NCT04273763) [47].

Favipiravir

Favipiravir is an antiviral drug approved for the treatment of influenza in Japan due to its activity against a wide spectrum of RNA viruses including coronaviruses [11]. This drug can effectively inhibit RdRp and has been reported to have activity against SARS-CoV-2 [4]. There are various ongoing trials with favipiravir and it has also been approved for use in multiple countries (ClinicalTrials.gov identifiers: NCT04351295, NCT04346628, NCT04392973, NCT04387760, NCT04425460, NCT04359615, NCT04445467, NCT04358549, NCT04349241, NCT04310228, NCT04373733, NCT04333589) [47].

Table 3 provides a summary of ongoing randomized control trials for emerging drugs. All information regarding the trials has been accessed from the ClinicalTrials.gov database [47].

**Table 3:** Randomized control trials for emerging drugs NCT ID: National Clinical Trial Identifier; oSOC: optimized standard of care; NET: neutrophil extracellular trap

Intervention	NCT ID	No. of participants		Duration		Phase	Sponsor
Stem cells							
Biological: PLX-PAD; biological: placebo	NCT04389450		140	June 2020–September 2021	Phase 2		Pluristem Ltd.
Biological: MultiStem; biological: placebo	NCT04367077		400	April 2020–August 2022	Phase 2, phase 3		Athersys, Inc
Targeting NETs							
Drug: dornase alfa inhalation solution (Pulmozyme)	NCT04359654		50	May 2020–November 2020	Phase 2		University College, London
Drug: anakinra 149 MG/ML prefilled syringe (Kineret)	NCT04443881		180	May 2020–March 2021	Phase 2, phase 3		Fundacion Miguel Servet
Drug: anakinra plus oSOC; drug: oSOC	NCT04364009		240	April 2020–September 2020	Phase 3		University Hospital, Tours
Drug: anakinra	NCT04341584		240	April 2020–December 2020	Phase 2		Assistance Publique - Hôpitaux de Paris
Drug: standard treatment; drug: oral administration of colchicine plus herbal phenolic monoterpene fractions	NCT04392141		200	April 2020–October 2020	Phase 1, phase 2		Kermanshah University of Medical Sciences
Drug: colchicine 1 MG oral tablet	NCT04375202		308	April 2020–October 2020	Phase 2		University Of Perugia
Drug: colchicine; other: local standard of care	NCT04328480		2500	April 2020–August 2020	Phase 3		Estudios Clínicos Latino América
Drug: colchicine plus symptomatic treatment (paracetamol); drug: symptomatic treatment (paracetamol or best symptomatic treatment based on doctor recommendations)	NCT04416334		1028	May 2020–December 2020	Phase 3		Instituto de Investigación Marqués de Valdecilla
Drug: colchicine	NCT04322565		310	April 2020–July 2020	Phase 2		Azienda Ospedaliero-Universitaria di Parma
Drug: colchicine; drug: placebo oral tablet	NCT04322682		6000	March 2020–September 2020	Phase 3		Montreal Heart Institute
Darunavir							
Drug: darunavir and cobicistat	NCT04252274		30	January 2020–December 2020	Phase 3		Shanghai Public Health Clinical Center
Favipiravir							
Drug: favipiravir; other: placebo	NCT04336904		100	March 2020–July 2020	Phase 3		Giuliano Rizzardini
Drug: favipiravir; drug: standard of care	NCT04434248		330	April 2020–July 2020	Phase 2, phase 3		Chromis LLC
Arbidol							
Drug: carrimycin; drug: lopinavir/ritonavir tablets or Arbidol or chloroquine phosphate; drug: basic treatment	NCT04286503		520	February 2020–February 2021	Phase 4		Beijing YouAn Hospital
Drug: Arbidol; other: basic treatment	NCT04260594		380	February 2020–December 2020	Phase 4		Jieming QU

## Conclusions

The COVID-19 pandemic has given rise to a public health crisis resulting in an extensive search for curative treatments. Research findings and recommendations are constantly evolving as various aspects of the virus and its transmission are being studied. Apart from the recently approved RDV, there are no other approved specific drugs that can cure patients infected by the virus. It has also been suggested that there is a possibility of a large number of undocumented asymptomatic infections, which facilitates the rapid spread of the virus. This is why the containment of the virus will be a particularly challenging task. Social distancing, contact tracing, and self-isolation are important strategies to avoid the spread of COVID-19 infection as mentioned by the Centers for Disease Control and Prevention (CDC). Many drugs listed in this review are encouraging and the benefits of these most likely outweigh the risk of adverse events and could potentially be life-saving. In addition, many coronaviruses have a highly mutable single-stranded RNA genome. Discovering new drugs against the virus is going to be challenging owing to the possible viral genetic recombinations. Extensive research is still needed to safely advocate the efficacy of the currently available therapeutic options.
